# *N*,*N*′-Bis(4-bromo­benzyl­idene)biphenyl-2,2′-diamine

**DOI:** 10.1107/S1600536809000993

**Published:** 2009-01-14

**Authors:** Saeed Dehghanpour, Saeedeh Asadizadeh, Shan Gao, Seik Weng Ng

**Affiliations:** a Department of Chemistry, Alzahra University, Vanak, Tehran, Iran; b School of Chemistry and Materials Science, Heilongjiang University, Harbin 150080, People’s Republic of China; c Department of Chemistry, University of Malaya, 50603 Kuala Lumpur, Malaysia

## Abstract

The complete molecule of the title Schiff base, C_26_H_18_Br_2_N_2_, is generated by crystallographic twofold symmetry. The aromatic rings of the biphenyl­ene portion of the mol­ecule are twisted, as shown by the dihedral of 61.8 (1)° formed between them.

## Related literature

There are relatively few crystallographic reports of Schiff bases formed by condensing biphenyl-2,2′-diamine with aldehydes or ketones. See: Alajarín *et al.* (2007 ▶); Coxall *et al.* (2003 ▶); Cunningham *et al.* (2004 ▶); Finder *et al.* (1973 ▶); Pruszynski *et al.* (1992 ▶).
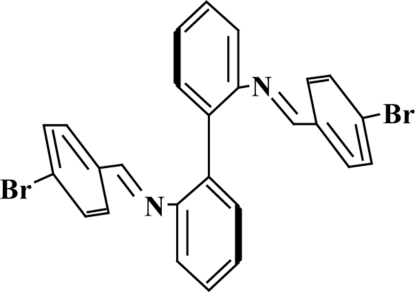


## Experimental

### 

#### Crystal data

C_26_H_18_Br_2_N_2_
*M* *_r_* = 518.24
Orthorhombic, 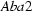

*a* = 15.9691 (10) Å
*b* = 8.3482 (5) Å
*c* = 16.7767 (11) Å
*V* = 2236.6 (2) Å^3^
*Z* = 4
Mo *K*α radiation
μ = 3.64 mm^−1^
*T* = 295 (2) K
0.28 × 0.25 × 0.19 mm

#### Data collection

Rigaku R-AXIS RAPID diffractometer
Absorption correction: multi-scan (*ABSCOR*; Higashi, 1995 ▶) *T*_min_ = 0.429, *T*_max_ = 0.545 (expected range = 0.394–0.501)
10424 measured reflections
2542 independent reflections
1333 reflections with *I* > 2σ(*I*)
*R*_int_ = 0.040

#### Refinement

*R*[*F* ^2^ > 2σ(*F* ^2^)] = 0.034
*wR*(*F* ^2^) = 0.116
*S* = 0.98
2542 reflections
136 parameters
1 restraint
H-atom parameters constrained
Δρ_max_ = 0.28 e Å^−3^
Δρ_min_ = −0.36 e Å^−3^
Absolute structure: Flack (1983 ▶), 1209 Friedel pairs
Flack parameter: −0.013 (15)

### 

Data collection: *RAPID-AUTO* (Rigaku, 1998 ▶); cell refinement: *RAPID-AUTO*; data reduction: *CrystalStructure* (Rigaku/MSC, 2002 ▶); program(s) used to solve structure: *SHELXS97* (Sheldrick, 2008 ▶); program(s) used to refine structure: *SHELXL97* (Sheldrick, 2008 ▶); molecular graphics: *X-SEED* (Barbour, 2001 ▶); software used to prepare material for publication: *publCIF* (Westrip, 2009 ▶).

## Supplementary Material

Crystal structure: contains datablocks I, global. DOI: 10.1107/S1600536809000993/tk2356sup1.cif

Structure factors: contains datablocks I. DOI: 10.1107/S1600536809000993/tk2356Isup2.hkl

Additional supplementary materials:  crystallographic information; 3D view; checkCIF report

## Figures and Tables

**Fig. 1.**
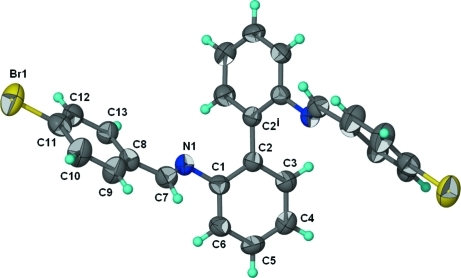
Thermal ellipsoid plot (Barbour, 2001); displacement ellipsoids are drawn at the 50% probability level, and H atoms as spheres of arbitrary radius. (Symmetry code: i = 2 - x, 3 - y, z).

## supplementary crystallographic information

### Experimental

Biphenyl-2,2'-diamine (5 mmol) and 4-bromobenzaldehyde (10 mmol) were dissolved in ethanol (50 ml). The solution was heated for 5 h; the solid that separated from the cooled solution was collected and recrystallized from chloroform; a second recrystallization was effected with ethanol. The yield as 90%. Analysis found: C 60.20, H 3.54, N 5.43; C_26_H_18_Br_2_N_2_ requires: C 60.26, H 3.50, N 5.41.

### Refinement

Carbon-bound H atoms were placed in calculated positions [C—H 0.93 Å and *U*_iso_(H) 1.2–1.5*U*_eq_(C)] and were included in the refinement in the riding-model approximation.

### Crystal data

**Table:**

C_26_H_18_Br_2_N_2_	*F*(000) = 1032
*M**_r_* = 518.24	*D*_x_ = 1.539 Mg m^−^^3^
Orthorhombic, *A**b**a*2	Mo *K*α radiation, λ = 0.71073 Å
Hall symbol: A 2 -2ac	Cell parameters from 5898 reflections
*a* = 15.9691 (10) Å	θ = 3.0–27.4°
*b* = 8.3482 (5) Å	µ = 3.64 mm^−^^1^
*c* = 16.7767 (11) Å	*T* = 295 K
*V* = 2236.6 (2) Å^3^	Cuboid, light yellow
*Z* = 4	0.28 × 0.25 × 0.19 mm

### Data collection

**Table:**

Rigaku R-AXIS RAPID diffractometer	2542 independent reflections
Radiation source: fine-focus sealed tube	1333 reflections with *I* > 2σ(*I*)
graphite	*R*_int_ = 0.040
Detector resolution: 10.000 pixels mm^-1^	θ_max_ = 27.4°, θ_min_ = 3.0°
ω scans	*h* = −18→20
Absorption correction: multi-scan (*ABSCOR*; Higashi, 1995)	*k* = −10→10
*T*_min_ = 0.429, *T*_max_ = 0.545	*l* = −21→21
10424 measured reflections	

### Refinement

**Table:**

Refinement on *F*^2^	Secondary atom site location: difference Fourier map
Least-squares matrix: full	Hydrogen site location: inferred from neighbouring sites
*R*[*F*^2^ > 2σ(*F*^2^)] = 0.034	H-atom parameters constrained
*wR*(*F*^2^) = 0.116	*w* = 1/[σ^2^(*F*_o_^2^) + (0.0547*P*)^2^] where *P* = (*F*_o_^2^ + 2*F*_c_^2^)/3
*S* = 0.98	(Δ/σ)_max_ = 0.001
2542 reflections	Δρ_max_ = 0.28 e Å^−^^3^
136 parameters	Δρ_min_ = −0.36 e Å^−^^3^
1 restraint	Absolute structure: Flack (1983), 1209 Friedel pairs
Primary atom site location: structure-invariant direct methods	Flack parameter: −0.013 (15)

### Fractional atomic coordinates and isotropic or equivalent isotropic displacement parameters (Å^2^)

**Table:**

	*x*	*y*	*z*	*U*_iso_*/*U*_eq_	
Br1	0.89090 (4)	0.63217 (6)	0.50003 (6)	0.1058 (3)	
N1	0.8979 (2)	1.3119 (4)	0.7320 (3)	0.0604 (9)	
C1	0.8877 (2)	1.4642 (5)	0.7689 (3)	0.0555 (10)	
C2	0.9576 (2)	1.5394 (4)	0.8036 (2)	0.0537 (9)	
C3	0.9461 (3)	1.6846 (5)	0.8415 (3)	0.0650 (11)	
H3	0.9920	1.7371	0.8636	0.078*	
C4	0.8672 (3)	1.7534 (6)	0.8471 (4)	0.0676 (13)	
H4	0.8605	1.8514	0.8727	0.081*	
C5	0.7989 (3)	1.6768 (5)	0.8150 (3)	0.0685 (12)	
H5	0.7459	1.7214	0.8205	0.082*	
C6	0.8087 (2)	1.5347 (5)	0.7748 (3)	0.0656 (12)	
H6	0.7626	1.4854	0.7514	0.079*	
C7	0.8654 (3)	1.2832 (7)	0.6649 (3)	0.0633 (12)	
H7	0.8381	1.3663	0.6387	0.076*	
C8	0.8685 (3)	1.1275 (5)	0.6264 (3)	0.0597 (11)	
C9	0.8443 (3)	1.1094 (5)	0.5480 (3)	0.0818 (14)	
H9	0.8236	1.1973	0.5202	0.098*	
C10	0.8505 (3)	0.9629 (6)	0.5101 (4)	0.0891 (14)	
H10	0.8346	0.9518	0.4570	0.107*	
C11	0.8805 (3)	0.8339 (6)	0.5523 (3)	0.0714 (13)	
C12	0.9015 (3)	0.8464 (5)	0.6305 (3)	0.0703 (13)	
H12	0.9195	0.7566	0.6585	0.084*	
C13	0.8961 (2)	0.9926 (5)	0.6683 (3)	0.0646 (11)	
H13	0.9108	1.0016	0.7217	0.078*	

### Atomic displacement parameters (Å^2^)

**Table:**

	*U*^11^	*U*^22^	*U*^33^	*U*^12^	*U*^13^	*U*^23^
Br1	0.1445 (6)	0.0704 (3)	0.1026 (5)	−0.0098 (2)	0.0284 (5)	−0.0131 (4)
N1	0.058 (2)	0.0512 (18)	0.072 (3)	−0.0048 (15)	0.0000 (19)	−0.0029 (19)
C1	0.050 (3)	0.058 (2)	0.058 (3)	0.0000 (18)	0.0025 (18)	0.009 (2)
C2	0.054 (2)	0.052 (2)	0.056 (2)	−0.0007 (16)	0.0007 (19)	0.0069 (19)
C3	0.063 (3)	0.064 (2)	0.067 (3)	−0.001 (2)	−0.005 (2)	−0.004 (2)
C4	0.081 (4)	0.057 (3)	0.065 (3)	0.004 (2)	−0.002 (3)	−0.002 (2)
C5	0.059 (3)	0.064 (2)	0.082 (3)	0.017 (2)	0.008 (2)	0.008 (2)
C6	0.052 (3)	0.065 (3)	0.080 (3)	−0.0025 (19)	0.000 (2)	0.014 (2)
C7	0.066 (3)	0.066 (3)	0.058 (3)	0.000 (2)	−0.007 (2)	0.010 (2)
C8	0.062 (2)	0.065 (3)	0.052 (3)	−0.0084 (18)	−0.002 (2)	0.001 (2)
C9	0.111 (4)	0.068 (3)	0.066 (3)	0.004 (3)	−0.020 (3)	0.002 (2)
C10	0.127 (4)	0.075 (3)	0.066 (3)	−0.001 (3)	−0.024 (4)	0.010 (3)
C11	0.065 (3)	0.084 (3)	0.065 (3)	−0.006 (2)	0.008 (2)	0.004 (3)
C12	0.070 (3)	0.058 (2)	0.083 (4)	−0.0047 (19)	−0.004 (3)	0.015 (2)
C13	0.074 (3)	0.060 (3)	0.059 (3)	0.001 (2)	−0.008 (2)	0.006 (2)

### Geometric parameters (Å, °)

**Table:**

Br1—C11	1.906 (5)	C6—H6	0.9300
N1—C7	1.262 (6)	C7—C8	1.452 (7)
N1—C1	1.424 (6)	C7—H7	0.9300
C1—C6	1.395 (5)	C8—C9	1.378 (7)
C1—C2	1.407 (6)	C8—C13	1.399 (6)
C2—C3	1.381 (6)	C9—C10	1.383 (6)
C2—C2^i^	1.506 (7)	C9—H9	0.9300
C3—C4	1.389 (6)	C10—C11	1.376 (7)
C3—H3	0.9300	C10—H10	0.9300
C4—C5	1.373 (7)	C11—C12	1.358 (8)
C4—H4	0.9300	C12—C13	1.378 (6)
C5—C6	1.374 (6)	C12—H12	0.9300
C5—H5	0.9300	C13—H13	0.9300
			
C7—N1—C1	120.7 (4)	N1—C7—H7	118.2
C6—C1—C2	120.0 (4)	C8—C7—H7	118.2
C6—C1—N1	120.8 (4)	C9—C8—C13	118.6 (4)
C2—C1—N1	119.2 (3)	C9—C8—C7	120.9 (4)
C3—C2—C1	118.5 (4)	C13—C8—C7	120.5 (5)
C3—C2—C2^i^	120.2 (4)	C8—C9—C10	121.1 (4)
C1—C2—C2^i^	121.2 (4)	C8—C9—H9	119.5
C2—C3—C4	121.0 (4)	C10—C9—H9	119.5
C2—C3—H3	119.5	C9—C10—C11	118.7 (5)
C4—C3—H3	119.5	C9—C10—H10	120.7
C5—C4—C3	120.1 (4)	C11—C10—H10	120.7
C5—C4—H4	120.0	C12—C11—C10	121.6 (5)
C3—C4—H4	120.0	C12—C11—Br1	119.4 (4)
C6—C5—C4	120.3 (4)	C10—C11—Br1	119.0 (4)
C6—C5—H5	119.8	C11—C12—C13	119.8 (5)
C4—C5—H5	119.8	C11—C12—H12	120.1
C5—C6—C1	120.1 (4)	C13—C12—H12	120.1
C5—C6—H6	119.9	C12—C13—C8	120.1 (5)
C1—C6—H6	119.9	C12—C13—H13	119.9
N1—C7—C8	123.6 (5)	C8—C13—H13	119.9
			
C7—N1—C1—C6	48.5 (6)	C1—N1—C7—C8	−175.6 (4)
C7—N1—C1—C2	−135.0 (5)	N1—C7—C8—C9	−169.2 (5)
C6—C1—C2—C3	−1.4 (6)	N1—C7—C8—C13	10.8 (7)
N1—C1—C2—C3	−177.9 (4)	C13—C8—C9—C10	−2.8 (8)
C6—C1—C2—C2^i^	175.4 (3)	C7—C8—C9—C10	177.2 (5)
N1—C1—C2—C2^i^	−1.1 (5)	C8—C9—C10—C11	0.6 (8)
C1—C2—C3—C4	1.6 (6)	C9—C10—C11—C12	2.1 (7)
C2^i^—C2—C3—C4	−175.3 (4)	C9—C10—C11—Br1	−179.0 (4)
C2—C3—C4—C5	0.2 (8)	C10—C11—C12—C13	−2.7 (7)
C3—C4—C5—C6	−2.2 (8)	Br1—C11—C12—C13	178.5 (3)
C4—C5—C6—C1	2.4 (7)	C11—C12—C13—C8	0.4 (7)
C2—C1—C6—C5	−0.6 (7)	C9—C8—C13—C12	2.2 (7)
N1—C1—C6—C5	175.9 (4)	C7—C8—C13—C12	−177.8 (4)

Symmetry codes: (i) −*x*+2, −*y*+3, *z*.

## References

[bb1] Alajarín, M., Bonillo, B., Sánchez-Andrada, P., Vidal, Á. & Bautista, D. (2007). *J. Org. Chem.* **72**, 5863–5866. 10.1021/jo0704661 17583957

[bb2] Barbour, L. J. (2001). *J. Supramol. Chem.* **1**, 189–191.

[bb3] Coxall, R. A., Lindoy, L. F., Miller, H. A., Parkin, A., Parsons, S., Tasker, P. A. & White, D. J. (2003). *Dalton Trans.* pp. 55–64.

[bb4] Cunningham, D., Gilligan, K., Hannon, M., Kelly, K., McArdle, P. & O’Malley, A. (2004). *Organometallics*, **23**, 984–994.

[bb5] Finder, C. J., Newton, M. G. & Allinger, N. L. (1973). *J. Chem. Soc. Perkin Trans. 2*, pp. 1929–1932.

[bb6] Flack, H. D. (1983). *Acta Cryst.* A**39**, 876–881.

[bb7] Higashi, T. (1995). *ABSCOR* Rigaku Corporation, Tokyo, Japan.

[bb8] Pruszynski, P., Leffek, K. T., Borecka, B. & Cameron, T. S. (1992). *Acta Cryst.* C**48**, 1638–1641.

[bb9] Rigaku (1998). *RAPID-AUTO* Rigaku Corporation, Tokyo, Japan.

[bb10] Rigaku/MSC (2002). *CrystalStructure* Rigaku/MSC, The Woodlands, Texas, USA.

[bb11] Sheldrick, G. M. (2008). *Acta Cryst.* A**64**, 112–122. 10.1107/S0108767307043930 18156677

[bb12] Westrip, S. P. (2009). *publCIF* In preparation.

